# A Locus Controlling Leaf Rolling Degree in Wheat under Drought Stress Identified by Bulked Segregant Analysis

**DOI:** 10.3390/plants11162076

**Published:** 2022-08-09

**Authors:** Xi Yang, Jingyi Wang, Xinguo Mao, Chaonan Li, Long Li, Yinghong Xue, Liheng He, Ruilian Jing

**Affiliations:** 1 College of Agronomy, Shanxi Agricultural University, Jinzhong 030801, China; 2 National Key Facility for Crop Gene Resources and Genetic Improvement, Institute of Crop Sciences, Chinese Academy of Agricultural Sciences, Beijing 100081, China

**Keywords:** *Triticum aestivum* L., drought, leaf rolling, bulked segregant analysis, gene mapping

## Abstract

Drought stress frequently occurs, which seriously restricts the production of wheat (*Triticum aestivum* L.). Leaf rolling is a typical physiological phenomenon of plants during drought stress. To understand the genetic mechanism of wheat leaf rolling, we constructed an F_2_ segregating population by crossing the slight-rolling wheat cultivar “Aikang 58” (AK58) with the serious-rolling wheat cultivar ″Zhongmai 36″ (ZM36). A combination of bulked segregant analysis (BSA) with Wheat 660K SNP Array was used to identify molecular markers linked to leaf rolling degree. A major locus for leaf rolling degree under drought stress was detected on chromosome 7A. We named this locus *LEAF ROLLING DEGREE 1* (*LERD1*), which was ultimately mapped to a region between 717.82 and 720.18 Mb. Twenty-one genes were predicted in this region, among which the basic helix-loop-helix (bHLH) transcription factor *TraesCS7A01G543300* was considered to be the most likely candidate gene for *LERD1*. The TraesCS7A01G543300 is highly homologous to the *Arabidopsis* ICE1 family proteins ICE/SCREAM, SCREAM2 and bHLH093, which control stomatal initiation and development. Two nucleotide variation sites were detected in the promoter region of *TraesCS7A01G543300* between the two wheat cultivars. Gene expression assays indicated that *TraesCS7A01G543300* was higher expressed in AK58 seedlings than that of ZM36. This research discovered a candidate gene related to wheat leaf rolling under drought stress, which may be helpful for understanding the leaf rolling mechanism and molecular breeding in wheat.

## 1. Introduction

Wheat (*Triticum aestivum* L.) is a staple food for more than 250 million people worldwide [1]. In addition to the benefit for nutrition and health, wheat provides about 21% of dietary calories and 20% of protein for humans, playing a prominent role in improving food security [2]. However, drought stress occurs frequently, which seriously restricts the production of wheat [3]. Therefore, improving wheat drought tolerance is an important approach to ensure food security [4,5]. By selecting and pyramiding favorable alleles related to drought tolerance traits in elite cultivars, crop performance in drought environments can be improved [6].

Leaf rolling is a typical physiological phenomenon of plants during drought stress, which is observed in various higher plants [7]. Leaf rolling is considered an adaptation to arid environments in wheat [8]. Moderate leaf rolling and erect leaf morphology are propitious to enhancing light capture, gas exchange for photosynthesis and carbon fixation [9]. Moreover, semi-rolling of leaves can reduce water loss by transpiration and interception of solar radiation by canopy; thus, it is of great significance to improve the adaptability of plants to environmental stress [10]. Consequently, the moderate rolling leaf trait is one of the aims of genetic improvement and molecular breeding in crops. Until now, more than 17 leaf rolling mutants, at least 70 genes/QTLs and 28 differentially expressed proteins related to leaf rolling traits have been reported in rice [11,12,13,14,15,16]. To date, at least five mutants with rolling leaves have been characterized in maize [17]. However, the genetic mechanism of leaf rolling under drought stress in wheat is rarely reported.

The stomatal is the main channel for water transpiration and gas exchange in plants, and changes in stomata density and morphology may affect plant drought tolerance [18]. An analysis of wilting mutant *multi-trait weakened* (*muw*) suggested that the increase in stomatal density can accelerate plant water loss and thus reduce drought tolerance [19]. In tomato, DELLA protein PROCERA (PRO) promotes the stomatal response to abscisic acid (ABA), and the pro mutant exhibited increased stomatal conductance and reduced drought tolerance [20]. Some proteins that are independent of the ABA signaling pathway can also play a function in drought tolerance by regulating stomatal closure, such as stress-responsive NAC 1 and SIMILAR TO RADICAL-INDUCED CELL DEATH1 [21,22].

In *Arabidopsis*, a genetic regulatory network has been identified that strictly controls stomatal development and pattern formation [23]. This includes three basic helix-loop-helix (bHLH) transcription factors that promote stomatal formation, SPEECHLESS (SPCH) is essential for the initiation of stomatal lineage, MUTE influences meristemoid to guard mother cell (GMC) conversion, FAMA determines the GMC to mature guard cell (GC) transition [24,25,26]. Two para-homologous protein ICE1/SCREAM (SCRM) and SCRM2 directly interact with SPCH, MUTE and FAMA, and then specify their sequential actions [27]. To prevent adjacent cells from becoming stomata, some extracellular and plasma membrane binding proteins are necessary to concert signals between developing stomatal and pavement cells [28], such as the EPIDERMAL PATTERNING FACTOR (EPF) and the Leu-rich repeat membrane protein TOO MANY MOUTHS (TMM) [29,30].

The fundamental mechanisms of stomatal formation in terrestrial plants are relatively conservative [23]. In rice, OsSPCH, OsMUTE, OsFAMA and OsICE1 are essential for the formation of mature stomata, which are orthologues of stomatal development regulators in Arabidopsis [31,32]. In moss, the most ancient extant stomata lineages, the formation of mature stomata requires PpSMF1 and PpSCRM1, which are also orthologous to the SPCH, MUTE, FAMA and ICE/SCRM in Arabidopsis [33,34]. The partnerships between ICE1/SCRM and SPCH, MUTE and FAMA are essential for the initiation and maturation of monocotyledonous stomata, but their protein function may be slightly different from that of Arabidopsis [31,35]. For example, in Brachypodium, the initiation of stomatal lineage requires the BdSCRM1, while the differentiation and function of stomatal complexes require BdSCRM2, which appears to be redundant in Arabidopsis [31,36]. A transcription factor BdMUTE has been proved to be necessary for subsidiary cell formation in the wheat relative Brachypodium [37].

The present study was based on the difference in leaf rolling degree under drought stress between two wheat cultivars, “Aikang 58” and “Zhongmai 36”. We constructed an F2 population by crossing the two cultivars and used a combination of BSA with a Wheat 660K SNP array to identify molecular markers linked to leaf rolling degree. The objective of the present study was to map the locus that controls leaf rolling in wheat under drought stress.

## 2. Results

### 2.1. The Leaf Rolling Degree of AK58 Was Lower than That of ZM36 under Drought Stress

**Figure 1:**
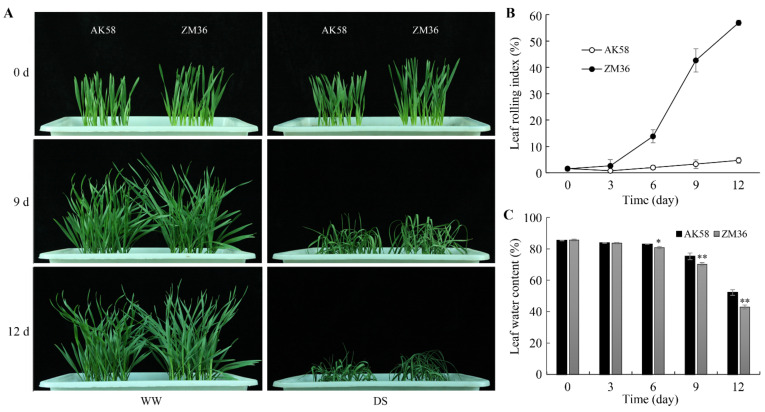
Under drought stress, the leaf rolling of ZM36 was more serious than that of AK58. (**A**) The seedling phenotype of the AK58 and ZM36 under WW (well-watered) and DS (drought stress) for 0, 9 and 12 d. (**B**) Leaf rolling index of AK58 and ZM36 under DS. (**C**) Leaf water contents of AK58 and ZM36 under DS. Data represent means ± SE. Error bars indicate SE. *, *t*-test with *p* < 0.05; **, *t*-test with *p* < 0.01.

Two Chinese wheat cultivars, “Aikang 58” (AK58) and “Zhongmai 36” (ZM36), were used to compare the difference in leaf rolling degree. AK58 is a high-yield variety widely cultivated in irrigated areas of China, released by the Henan Institute of Science and Technology in 2005. ZM36 is a stable-yield variety suitable for rain-fed area cultivation, released by the Institute of Crop Sciences, Chinese Academy of Agricultural Sciences in 2018. The plants of AK58 and ZM36 grew well under well-watered conditions. On the 9th day of drought treatment, AK58 plants exhibited flat leaves, while ZM36 plant leaves showed a slight rolling phenotype. On the 12th day of drought treatment, the difference in leaf morphology became more obvious: the leaves of AK58 were wilted but remained flat, and the leaves of ZM36 were wilted and seriously rolled (Figure 1A). With the increase in drought stress treatment time, the leaf rolling index (LRI) of ZM36 leaves increased significantly, while AK58 leaves did not change significantly (Figure 1B). The leaf water contents of AK58 and ZM36 decreased; however, from the 6th day, the leaf water content of ZM36 was significantly lower than that of AK58 (Figure 1C). Therefore, wheat variety AK58 was identified as ″slight-rolling″ type, while ZM36 was “serious-rolling” type under drought stress at the seedling stage.

### 2.2. AK58 Is More Tolerant to Drought than ZM36 at Seedling Stage

**Figure 2:**
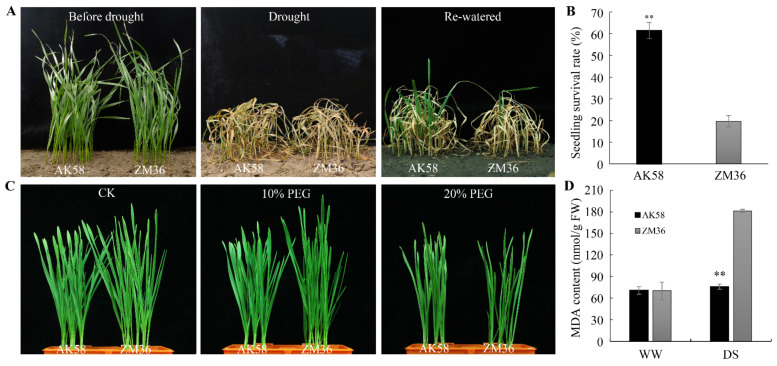
Seedlings of wheat cultivar AK58 were more tolerant to dehydration than ZM36. (**A**) Performance of AK58 and ZM36 seedlings before drought (left), drought stress (DS) for 18 days (middle) and re-watered for 11 days (right). (**B**) Seedling survival rates of AK58 and ZM36 exposed to drought stress followed by re-watering. (**C**) Seedlings of AK58 and ZM36 grown in hydroponic culture under water (CK) or PEG-6000 treatment for 36 h. (**D**) MDA contents in leaves of AK58 and ZM36 seedlings grown under well-watered (WW) and DS. Values represent means ± SE. **, *t*-test with *p* < 0.01.

To investigate the relationship between seedling drought tolerance and leaf rolling degree in wheat, we evaluated the seedling drought tolerance of ZM36 and AK58. On the 11th day after re-watering, the survival rate of AK58 plants was 61.4%, and that of ZM36 was 19.6% (Figure 2A,B). The Malondialdehyde (MDA) content of ZM36 was significantly higher than that of AK58 on the 9th day of drought treatment (Figure 2D). We also performed PEG-600 treatment to simulate drought stress. Both AK58 and ZM36 plants exhibited turgid and flat leaves in the medium without PEG-6000 (Figure 2C left), but in mediums with 10% or 20% PEG-6000, ZM36 plants showed a rolling-leaf phenotype, while AK58 leaves remained flat (Figure 2C). These results demonstrated that AK58 has stronger drought tolerance than ZM36 at the seedling stage. In this experiment, the drought tolerance of wheat cultivars AK58 and ZM36 at the seedling stage was negatively correlated with the leaf rolling degree under drought stress.

### 2.3. AK58 Leaves Exhibited Lower Stomatal Density than That of ZM36

**Figure 3:**
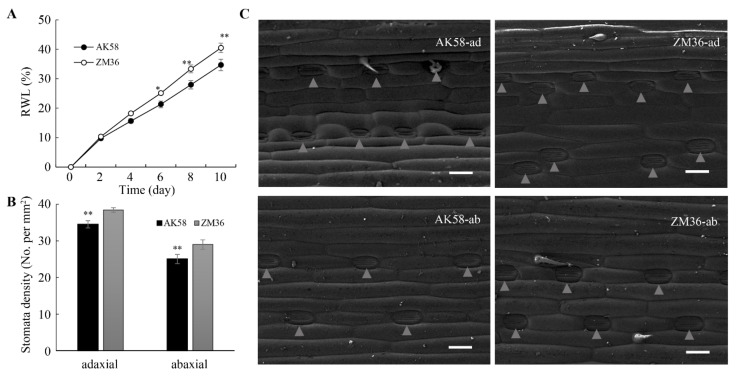
ZM36 has a higher leaf RWL and stomatal density than AK58. (**A**) RWL of AK58 and ZM36 plants. (**B**) Stomata density of AK58 and ZM36. Data represent means ± SE. *, *t*-test with *p* < 0.05; **, *t*-test with *p* < 0.01. (**C**) SEM analysis of the adaxial (ad) and abaxial (ab) epidermis of AK58 and ZM36. Triangles indicate stomata. Scale bars = 100 μm.

To investigate the physiological basis of leaf rolling, we measured the rate of water loss (RWL) from excised-leaf of AK58 and ZM36. The RWL of ZM36 was significantly higher than that of AK58 from the 6th day (Figure 3A). Stomata on the leaf surface are the main channel for plants to discharge water [38]. Therefore, the difference in RWL between AK58 and ZM36 may be caused by the difference in stomatal morphology and/or density. There was no significant difference in the stomatal characteristics between AK58 and ZM36 leaves under drought stress (Figure S1). We then observed the leaf surface structures of AK58 and ZM36 plants by scanning electron microscopy (SEM). The stomatal densities on the adaxial (ad) or abaxial (ab) epidermis of ZM36 leaves were significantly higher than that of AK58 (Figure 3B,C); however, no other significant structural differences were observed on the AK58 and ZM36 leaf surface in this experiment.

### 2.4. Genetic Analysis and Mapping of Leaf Rolling Degree Locus

**Figure 4:**
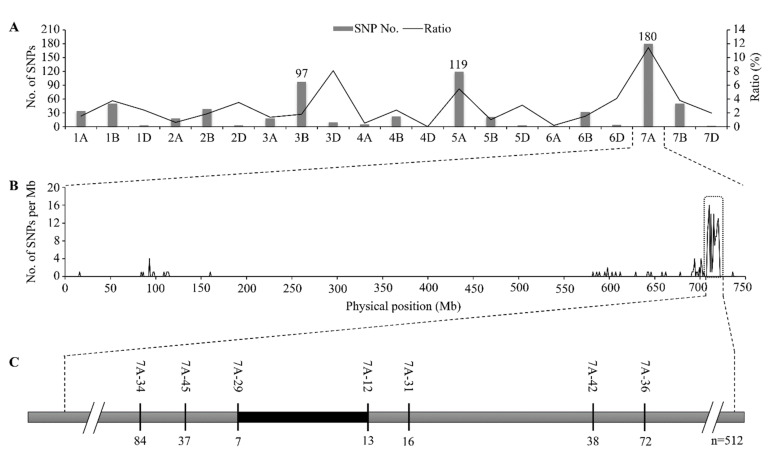
Mapping of locus controlling wheat leaf rolling degree. (**A**) The number and percentage of polymorphic SNP loci in each of the 21 wheat chromosomes. (**B**) The number of SNPs in each 1 Mb region of chromosome 7A identified using the Wheat 660K SNP array. (**C**) *LERD1* was mapped to a 2.36 Mb region between markers 7A-29 and 7A-12. The black rectangle represents the final target region harboring *LERD1*. The numbers below the bar indicate the number of recombinants between *LERD1* and the molecular marker.

To understand the genetics underlying the leaf rolling in wheat, we constructed a wheat F_2_ segregating population by crossing a slight-rolling type cultivar AK58 with a serious-rolling cultivar ZM36. Under drought stress, seedlings of F_2_ population individuals showed a continuous phenotypic distribution from slight-rolling to serious-rolling, which provided evidence that leaf rolling degree was a quantitative trait (Figure S2). A combination of BSA with Wheat 660K SNP Array was used to identify molecular markers linked to leaf rolling degree. Between the two parents and two bulks, a total of 721 SNP loci from the Wheat 660K SNP Array showed homozygous polymorphisms. The highest number and proportion of polymorphic SNPs were identified on chromosome 7A among all 21 chromosomes, indicating that 7A was likely the chromosome carrying the rolling degree locus, which was consequently named *LEAF ROLLING DEGREE 1* (*LERD1*) (Figure 4A). The chromosome interval 708–721 Mb was the predicted region for *LERD1* (Figure 4B).

To minimize the effects of errors caused by environmental effects and phenotyping errors, we chose 256 plants with serious-rolling phenotypes from the F_2_ population of ~4000 individuals for further analysis. Seven polymorphic molecular markers were used to genotype individuals in the mapping population. According to the number of recombinants between *LERD1* and the molecular markers in the mapping population, *LERD1* was ultimately mapped to a region between 7A-29 (717.82 Mb) and 7A-12 (720.18 Mb) on chromosome 7A (Figure 4C).

### 2.5. Candidate Gene Prediction and Bioinformatics Analysis

**Figure 5:**
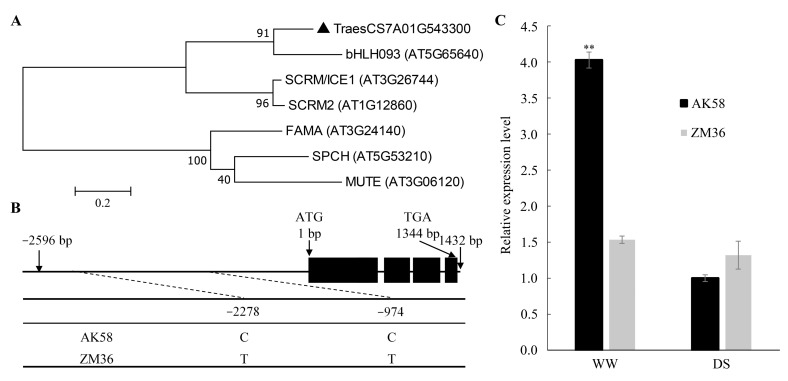
Candidate gene analysis. (**A**) Molecular phylogeny of TraesCS7A02G543300 and related bHLHs in *Arabidopsis*. (**B**) Schematic diagram of the *TraesCS7A02G543300* structure. The ATG start codon was designated as position 1 bp. Polymorphic sites were detected in the promoter region of *TraesCS7A02G543300*. (**C**) qPCR analysis of gene *TraesCS7A02G543300* expression. Data represent means ± SE (n = 3). **, *p* < 0.01.

A total of 21 genes were predicted in the target chromosome region, including a bHLH transcription factor TraesCS7A01G543300 (Table S1, Figure S3). TraesCS7A01G543300 encodes homologs of the ICE1/SCREAM, SCRM2 proteins and bHLH93 protein from Arabidopsis (Figure 5A), which were considered to be the major regulators of stomatal development in *Arabidopsis* [27]. Sequence polymorphism assays indicated that there was no nucleotide variation site in the coding region of *TraesCS7A01G543300*, but two SNPs were detected in the promoter region, one at −2278 bp and another at −974 bp between AK58 and ZM36 (Figure 5B). Differences in the promoter region may affect gene expression, so we measured the relative expression of genes at the seedling stage. The relative expression of *TraesCS7A01G543300* in AK58 seedling was 2.6 times that of ZM36 under the WW condition (Figure 5C). Therefore, *TraesCS7A01G543300* was considered the most likely candidate gene for *LERD1*.

## 3. Discussion

Traditional quantitative trait loci (QTL) mapping methods rely on phenotyping and genotyping of a large number of individuals from a mapping population, which is time-consuming and laborious [39]. BSA has been used to overcome this problem by only genotyping individuals with extreme phenotypes [40]. The advent and application of BSA and next-generation sequencing technologies have provided new opportunities for the rapid identification of QTLs [41]. Wang et al. developed a method to map QTLs by directly sequencing graded-pool samples from F_2_ progeny using modified BSA [42]. Yu et al. used an F_2_ population for Bulked Segregant RNA-seq (BSR-seq) and cloned a *knotted 1* homolog [43]. Wheat 660K SNP array is economical and reliable, which demonstrates great potential for marker-assisted selection [44]. In the present study, a combination of BSA and Wheat 660K SNP array was used to identify the locus affecting the leaf rolling of the wheat seedling. The *LERD1* has been mapped to a 2.36 Mb region, but further fine mapping requires more genetic populations, such as recombinant inbred lines (RIL) or near-isogenic lines (NIL) population.

Leaf rolling occurs when the uptake of soil water by the root system does not balance the need for evaporation [45]. Vascular plants maintain a balance between CO_2_ absorption and water loss by stomatal movement, about 90% of leaf water loss in plants occurs through stomata [46,47]. Under drought stress, plants protect themselves from excessive water loss by diminishing stomatal aperture to reduce transpiration [48]. Previous studies have shown that wheat varieties that can maintain comparatively high leaf water potential and low transpiration rate under drought conditions tend to have stronger drought tolerance [49]. Leaf rolling in wheat can improve fog capturing and transport and enhance adaptation to drought stress in an arid climate [50,51].

In this study, the leaf water content of ZM36 was significantly lower than that of AK58 under drought stress, and the RWL of ZM36 was higher than that of AK58 (Figure 1C and Figure 3A). We observed that there was no significant difference in stomatal aperture between AK58 and ZM36 under drought stress (Figure S1), while the stomatal density of ZM36 was higher than that of AK58 (Figure 3B,C). Therefore, we speculate the difference in RWL between ZM36 and AK58 is probably related to the difference in stomatal density. The candidate genes may be involved in the regulation of stomatal density in wheat plants, thereby affecting the water loss rate of wheat plants under drought stress. An environmentally induced leaf-rolling phenotype is usually caused by abnormalities in the number, morphology, size or distribution of the bulliform cells [10]. Whether the bulliform cells of AK58 and ZM36 are different needs to be further analyzed.

The bHLH093 belongs to the ICE1 family bHLH-LZs, the expression of *bHLH093* may competitively inhibit the function of SCRM [27]. Overexpression of *bHLH093* with the 35S promoter resulted in a weak decreased number of mature stomata phenotypes [26]. In this study, *TraesCS7A02G543300* mapped within the target chromosome region encodes a bHLH family protein homologous to SCRM and bHLH093, which is more evolutionarily similar to *bHLH093* (Figure S3). The AK58 seedlings with higher *TraesCS7A02G543300* expression had a relatively lower stomatal density, while ZM36 with lower gene expression and relatively higher stomatal density. Our results suggested that *TraesCS7A02G543300* may have similar functions as *bHLH093*, but further detailed experimental evidence is needed.

In this study, we mapped a locus that controls leaf rolling of wheat seedlings under drought stress. We named it *LERD1*, which was finally mapped to a 2.36 Mb region between 717.82 and 720.18 Mb on chromosome 7A. A phenotypic analysis of two parents indicated that the RWL of ZM36 was higher than that of AK58, and the stomatal density of ZM36 was significantly higher than that of AK58. The bHLH transcription factor *TraesCS7A01G543300*, which is an ortholog of stomatal development regulators in *Arabidopsis*, was considered to be the most likely candidate gene for *LERD1*.

## 4. Materials and Methods

### 4.1. Plant Materials and Growth Condition

Two Chinese wheat cultivars, “Aikang 58” (AK58) and “Zhongmai 36” (ZM36) and an F_2_ population derived from the cross of AK58 × ZM36 were used as the plant materials. AK58 is a high-yield cultivar widely cultivated in irrigated areas of China, released by the Henan Institute of Science and Technology in 2005 [52]. ZM36 is a stable-yield cultivar suitable for rain-fed area cultivation, released by the Institute of Crop Sciences, Chinese Academy of Agricultural Sciences in 2018. A total of about 4000 individuals of the F_2_ population were used for genetic analysis and fine mapping.

For the pot experiment, wheat seeds were sown in containers (length 26 cm, width 19 cm, and height 9 cm), each containing 3 kg of mixed loam and organic fertilizer. The containers were placed horizontally in the field under rain-off shelter, which is located in the National Wheat Improvement Center in Beijing, China (116°28′ E, 39°48′ N).

### 4.2. Leaf Rolling Degree Assays

The leaf phenotype of wheat seedlings was observed under well-watered (WW) and drought stress (DS) treatments. WW refers to maintaining an adequate water supply, and DS means stop watering from the three-leaf stage. The day of the last watering was defined as the 0th day of drought treatment. On the 0th, 9th and 12th day of drought treatment, the soil moisture content was 21.8%, 7.2% and 5.8%, respectively.

For LRI (leaf rolling index), the largest leaf width (Lw) and the natural distance of the leaf margins (Ln) of the second fully expanded leaves were measured during 09:00–10:30. LRI was calculated using a formula LRI = (Lw − Ln)/Lw × 100% [53]. The experiment was set up in three biological replicates, and 10 leaves were measured in each replicate.

For leaf water content, fresh leaf weight (Fw) was measured immediately after sampling. Dry weight (Dw) was recorded after drying the leaves at 70 °C. Leaf water content was calculated as (Fw − Dw)/Fw × 100%.

According to the leaf morphology of plants treated with drought for 12 d, the leaf rolling degree of the F_2_ population was classified into three grades: slight-rolling, leaf blade remained flat; moderate-rolling, leaf blade is partially contracted and curled; serious-rolling, leaf blade curled tightly (Figure S2).

### 4.3. Drought Tolerance Assays

For seedling survival rate, 14-day-old plants were stopped from water supply for 18 d, then re-watered, and the number of surviving plants was surveyed on the 11th day. On the 0th and 18th day of drought treatment, the soil moisture content was 21.8% and 3.9%, respectively. Statistical data were based on data obtained from three independent experiments.

For osmotic stress, 7-day-old seedlings were cultured in a hydroponic medium containing different concentrations of polyethylene glycol (PEG-6000) for 48 h. The plants were cultured in growth chambers with a 14 h light: 10 h dark period, 25 °C: 20 °C, and 70% relative humidity.

### 4.4. Rate of Water Loss (RWL) from Excised-Leaf

For detection of RWL, about 5 g of excised fresh leaves were incubated in the dark at 25 °C with 70% relative humidity and weighed every 2 h. RWL = (W_0_ − W*_t_*)/W_0_. W_0_: fresh weight of initial leaves. W*_t_*: weight of leaves at *t* hours. The experiment was set up in three biological replicates.

### 4.5. Leaf Structure Assays

For light microscope analysis, the leaves of AK58 and ZM36 seedlings on the same leaf position were selected for stomatal aperture assay on the 5th day of drought treatment. The brush was dipped in gum Arabic solution and applied on the surface of leaves at 10:00. After drying, the film was removed with tweezers and placed on the slide, and one drop of distilled water was added to prepare the film and examined for stomatal opening using a light microscope (B5–223 IEP, Motic China Group, Xiamen, China).

For scanning electron microscopy (SEM) analysis, the leaves with the same position were collected from the three-leaf seedlings. Leaf samples were fixed on the sample table using conductive tape before being imaged by SEM (TM4000, Hitachi, Japan) at 5 kV.

### 4.6. Genetic Analysis of Leaf Rolling

The chromosome loci linked to leaf rolling was identified using the bulked segregant analysis (BSA) method, following the procedure of Wu et al. [54]. Two pools of extreme types of leaf rolling were constructed using the F_2_ population. The slight-rolling pool was prepared by mixing equal amounts of leaves sampled from 20 individuals with slight-rolling. The serious-rolling pool was prepared by mixing equal amounts of leaves sampled from 20 serious-rolling individuals.

Genomic DNA was isolated from leaves using the CTAB method [55]. The two pools and two parents were genotyped with the Wheat 660K SNP Array (CapitalBio Technology Corporation, Beijing, China). The monomorphic, heterozygous and poor-quality SNP markers with ambiguous SNP signals were excluded, and homozygous polymorphism SNP markers linked to leaf rolling degree were used for further analysis. The physical locations of all SNP markers were searched in the IWGSC RefSeq v1.0, using BLAST.

### 4.7. Fine Mapping

To finely map the target locus, 18 SSR markers and 14 InDel (insertion/deletion) markers were developed in the predicted chromosomal region, of which four SSR markers and three InDel markers were polymorphic between two parents (Table S2). The mapping population was constructed from 256 individuals with serious-rolling phenotypes selected from the F_2_ population of ~4000 individuals. The seven polymorphic markers and mapping population were used for genotyping analysis.

### 4.8. Bioinformatics Analysis and Expression Analysis of Candidate Gene

To predict functions of candidate genes, their amino acid sequences were used to blast against the NCBI database (https://www.ncbi.nlm.nih.gov/, accessed on 6 January 2021). The polymorphism assays of genomic DNA sequences were conducted using Lasergene 7.1.0 (DNASTAR, Inc., Madison, WI, USA). A neighbor-joining phylogenetic tree was constructed using the MEGA software v5.2 (https://www.megasoftware.net/, https://www.ncbi.nlm.nih.gov/, accessed on 6 January 2021).

Total RNA was extracted from young leaves of 15-day-old seedlings using an RNAprep Pure Plant Kit (Tiangen, Beijing, China) and reverse transcription was performed using a FastQuant RT Kit with gDNase (Tiangen, Beijing, China). Real-time quantitative RT-PCR was performed on a Roche LightCycler^®^ 96 Real-Time PCR System using the SYBR Green PCR Master Mix Reagent “Tli RNaseH Plus” (TaKaRa, Beijing, China) [56]. *TaTubulin* was used as the endogenous control. The primers are listed in Table S2.

## Data Availability

Not applicable.

## Supplementary Materials

The following supporting information can be downloaded at: https://www.mdpi.com/article/10.3390/plants11162076/s1, Figure S1: Stomatal morphology on the abaxial surface of AK58 and ZM36 leaves under drought stress; Figure S2: Leaf rolling degree of the F_2_ population; Figure S3. Molecular phylogeny of TraesCS7A02G543300 and related bHLHs; Table S1: Annotations of genes predicted in the candidate region on chromosome 7A; Table S2: Primers used in this study.
