# Supplementary material for: A Locus Controlling Leaf Rolling Degree in Wheat under Drought Stress Identified by Bulked Segregant Analysis
Source: Plants (Basel). 2022 Aug 9;11(16):2076. doi: 10.3390/plants11162076 (PMC9414355; doi:10.3390/plants11162076)
Supplement: Supplementary file 1 [file plants-11-02076-s001.zip › plants-1839286-supplementary.pdf]

The following are the supplementary data related to this article.

Figure S1. Stomatal morphology on the abaxial surface of AK58 and ZM36 leaves under drought stress.

Figure S2. Leaf rolling degree of the F_2 population.

Figure S3. Molecular phylogeny of TraesCS7A02G543300 and related bHLHs.

Table S1. Annotations of genes predicted in the candidate region on chromosome 7A.

Table S2. Primers used in this study.

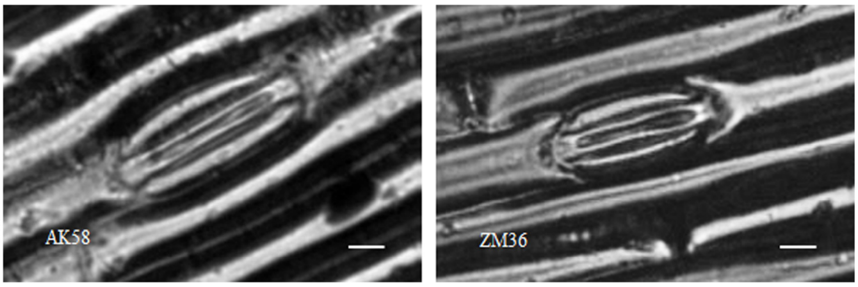

Figure S1. Stomatal morphology on the abaxial surface of AK58 and ZM36 leaves under drought stress. Scale bars=10 μm.

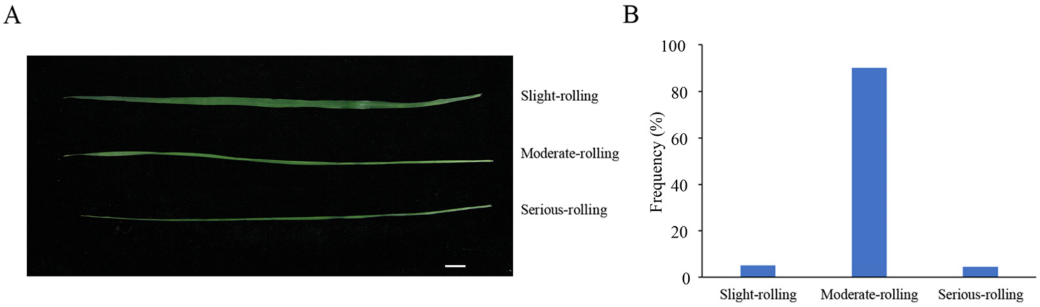

Figure S2. Leaf rolling degree of the F_2 population.

(A) Criteria for grading leaf rolling degree. (Scale bar, 1 cm.). (B) Frequency distribution of leaf rolling degree in the F_2 population derived from a cross of AK58×ZM36 under drought stress.

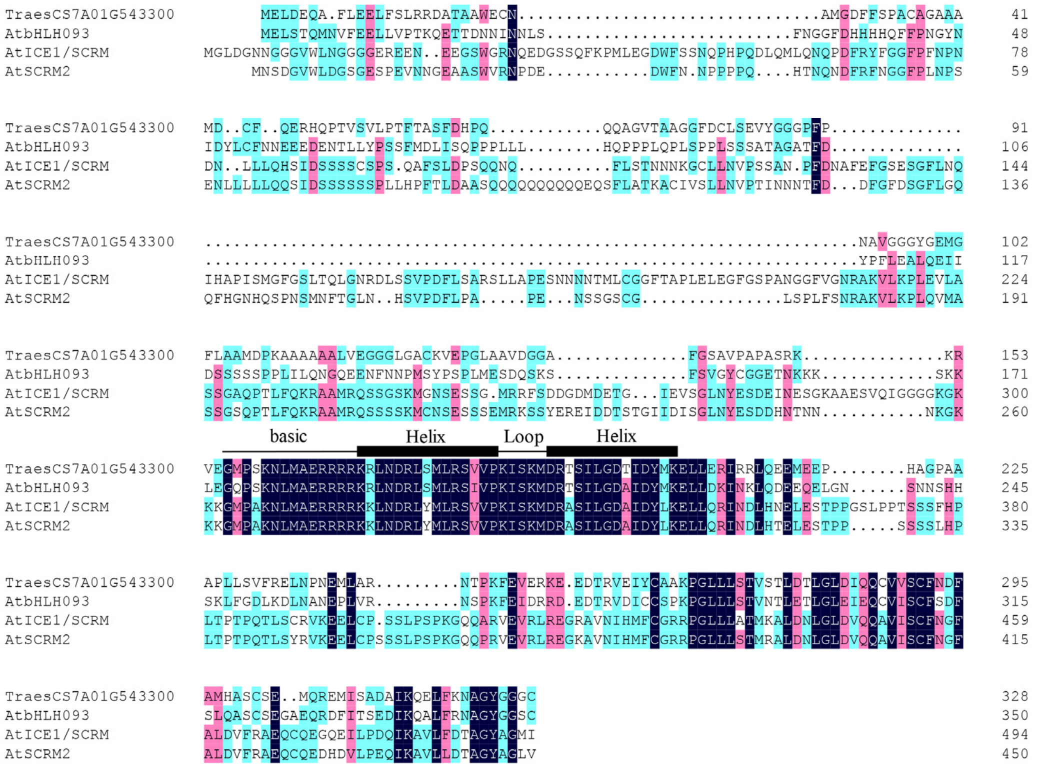

Figure S3. Amino acid sequence alignment of SCRM homologs.

Table S1. Annotations of genes predicted in the candidate region on chromosome 7A

Gene ID	Description
TraesCS7A01G541200	Protein kinase family protein
TraesCS7A01G541300	Disease resistance protein RPM1
TraesCS7A01G541400	receptor kinase 1
TraesCS7A01G541500	transmembrane protein, putative (DUF594)
TraesCS7A01G541600	Zinc finger-like protein
TraesCS7A01G541700	Zinc finger family protein
TraesCS7A01G541800	Zinc finger-like protein
TraesCS7A01G541900	Zinc finger-like protein
TraesCS7A01G542000	1,4-dihydroxy-2-naphthoyl-CoA synthase
TraesCS7A01G542100	Zinc finger-like protein
TraesCS7A01G542200	Zinc finger family protein
TraesCS7A01G542300	Zinc finger-like protein
TraesCS7A01G542400	Zinc finger-like protein
TraesCS7A01G542500	UPF0496 protein
TraesCS7A01G542600	Blue copper protein

TraesCS7A01G542700	Transcription factor
TraesCS7A01G542800	Lateral signaling target 2-like protein
TraesCS7A01G542900	DUF616 protein
TraesCS7A01G543000	DDB1-and CUL4-associated factor
TraesCS7A01G543100	DDB1-and CUL4-associated factor 8
TraesCS7A01G543200	transmembrane protein, putative (DUF594)
TraesCS7A01G543300	Basic helix-loop-helix transcription factor
TraesCS7A01G543400	Serine/threonine-protein kinase

The predicted genes and their annotations were retrieved from IWGSC (http://www.wheat genome.org/).

Table S2. Primers used in this study.

Primer set	Nucleotide sequence (5' to 3')	Purpose	physical location (Mb)
7A-34F	GCTAGCCTCTATTCTGAAGCGAT	SSR marker	716.39
7A-34R	GATGTCAGACGGGAACATGGTAT	SSR marker	716.39
7A-45F	AGTGCATTGAAATTTACCTGCCG	InDel marker	717.17
7A-45R	GGAGGAATTTGCATCTGTACAGC	InDel marker	717.17
7A-29F	CGGTTTCACAGCAGAAACAAAGCTACTG	InDel marker	717.82
7A-29R	GAAGTGTTTGAAGAAATGACAGACTCGGAC	InDel marker	717.82
7A-12F	GCCTTGACCTATGACCGTGTTA	SSR marker	720.17
7A-12R	AACAGAAATGTTGGATCGTTTGC	SSR marker	720.17
7A-31F	ATATATGGACAAAGAACAGGCGTGCG	InDel marker	720.88
7A-31R	CAATGTTAATCTCCGCGAAACCCCATA	InDel marker	720.88
7A-42F	TGGAGAGTGGTGGAAAGTGTAAT	SSR marker	721.59
7A-42R	TCCTTTCCTCCTTCTCTCTTCTCT	SSR marker	721.59
7A-36F	TCTGAACTTAAGTGAGAGCTGTT	SSR marker	722.44
7A-36R	GCAGAGGTAGGCAGATGGG	SSR marker	722.44
q543300F	TTCGACCACCCACAGCAGCA	qRT-PCR	No
q543300R	TCCACCCGCTTCTTCCTCGA	qRT-PCR	No
TaTubulinF	GAGGCCTCGTGTGGTCGCTTTGT	qRT-PCR	No
TaTubulinR	GCCCAGTTGTTACCCGCACCAGA	qRT-PCR	No
